# Preserving Sample, Interface, and Signal Fidelity in Wearable Sweat Electrolyte Monitoring During Exercise

**DOI:** 10.3390/bios16090523

**Published:** 2026-09-19

**Authors:** Qi Qi, Jinjie Duan, Yuning Lei, Shuang Zhou, Qian Liu, Qiong Wang, Nan Gao, Wenjuan Sun, Zhiming Zhan, Yuxiang Wu

**Affiliations:** 1 School of Optoelectronic Materials and Technology, Jianghan University, Wuhan 430056, China; qiqi@stu.jhun.edu.cn (Q.Q.); djjstudy@sina.com (J.D.); yuninglei@stu.jhun.edu.cn (Y.L.); shuangzhou@stu.jhun.edu.cn (S.Z.); 2 Institute of Intelligent Sport and Proactive Health, Department of Health and Physical Education, Jianghan University, Wuhan 430056, China; liuqian@stu.jhun.edu.cn (Q.L.); wangqiong@stu.jhun.edu.cn (Q.W.); gao2411@jhun.edu.cn (N.G.); 3 School of Artificial Intelligence, Jianghan University, Wuhan 430056, China; wjsun@jhun.edu.cn

**Keywords:** sweat electrolyte monitoring, wearable sweat sensors, ion-selective electrodes, exercise monitoring, microfluidics, system-level fidelity

## Abstract

Sweat electrolytes, including Na^+^, K^+^, and Cl^−^, provide dynamic information on exercise-induced fluid and electrolyte regulation. Wearable ion-selective electrode (ISE) platforms now enable continuous, in situ monitoring of these ions, but performance in standard solutions does not guarantee reliable on-body data. During exercise, measurement distortion can arise before sweat contacts the electrode, at the electrochemical interface, and during signal acquisition and interpretation. This review synthesizes these coupled distortion pathways through a three-layer fidelity framework. Sample fidelity describes whether collected sweat preserves its temporal sequence, concentration state, and spatial origin during collection and microfluidic transport. Interface fidelity concerns stable ion recognition, ion-to-electron transduction, and reference-potential output in complex sweat matrices under deformation. Signal fidelity covers potential readout, transmission, calibration, temperature compensation, drift correction, and concentration estimation. By organizing recent work around these layers, we identify how sampling lag, residual-sweat mixing, evaporation, fouling, component leaching, interfacial drift, reference instability, and algorithmic overprocessing can propagate into biased electrolyte profiles. We argue that wearable sweat electrolyte monitoring should move from isolated electrode optimization toward end-to-end fidelity preservation and validation under realistic exercise conditions. This framework provides a practical basis for sensor design, performance reporting, and translation to sports, occupational safety, and digital health applications.

## 1. Introduction

During exercise, sweat electrolyte dynamics provide one of the few noninvasive windows into fluid and electrolyte regulation at the skin surface [1,2]. Sodium, potassium, and chloride concentrations vary with sweat rate, exercise intensity, environmental heat load, regional sampling site, individual acclimation, and fluid intake [2,3,4]. In addition to these exercise-related factors, the mode of sweat induction can itself introduce variability in sampling conditions; for example, pharmacological stimulation with pilocarpine enables controlled, on-demand sweat generation under resting conditions, thereby providing more controlled conditions for sweat sampling [5]. Continuous monitoring of these ions could support hydration assessment, individualized rehydration strategies, heat-stress management, and exercise safety [6,7]. This promise has made sweat an attractive matrix for wearable biochemical sensing because it is accessible in situ and can be sampled without venipuncture [8,9]. However, the value of sweat electrolyte data depends on whether the measured signal still represents the current sweat reaching the device, rather than an altered sample produced by collection, transport, interfacial, or signal-processing artifacts [10,11,12,13].

Wearable sweat electrolyte sensors have developed through changes in sensing materials, device architectures, and system integration [10,14,15,16]. Early work by Joseph Wang and co-workers demonstrated the feasibility of electrochemical sensing directly on the skin through tattoo-based platforms for real-time sweat analysis during exercise and wireless potentiometric monitoring of sweat Na^+^ [17,18]. Complementary advances by John A. Rogers and co-workers established ultrathin epidermal electronic systems for conformal skin interfacing and later introduced soft skin-interfaced microfluidic platforms for sweat collection and biochemical analysis during physical activity [19,20]. As wearable sweat sensing developed, these advances in skin interfacing, sweat handling, electrochemical detection, and portable electronics increasingly converged within integrated devices. Within this broader development, potentiometric sensors based on ISEs have become a major route for sweat-ion monitoring because they are readily miniaturized, consume little power, and can support continuous measurement [12,21,22]. Further advances in ion-selective membranes (ISMs), solid-contact materials, flexible electronics, microfluidics, and wireless communication have moved sweat electrolyte analysis from ex situ testing toward in situ wearable monitoring [15,23,24]. Integrated platforms now combine sweat collection, ion recognition, potential readout, wireless transmission, and data display, providing a technical basis for continuous monitoring during exercise [14].

The central reliability problem is that a near-Nernstian slope in standard solutions does not ensure accurate, stable, or interpretable data during actual wear [12,13,25]. Standard-solution tests usually control temperature, ionic background, sample volume, and measurement environment. Exercise conditions, instead, introduce changing sweat rate, skin deformation, evaporation, fouling, temperature variation, mechanical perturbation, and wireless transmission instability. These factors can act across the full pathway from sweat secretion and transport to the sensing interface, electrical readout, transmission, and concentration interpretation [13,16]. Wearable sweat electrolyte monitoring therefore depends not only on electrode sensitivity, linear range, and selectivity, but also on whether the whole system preserves the integrity, continuity, and interpretability of electrolyte information under real exercise conditions [11,12,13].

Several review streams have shaped the current understanding of wearable sweat sensing. Reviews on sweat physiology and applications have clarified secretion mechanisms, biomarker availability, sweat–blood relationships, in vivo validation needs, and clinical translation barriers [26,27,28]. Device-focused reviews have summarized flexible electronics, sensing materials, electrochemical and non-electrochemical detection modes, wireless transmission, power supply, and multiparameter integration [29,30,31]. System-level reviews have emphasized sweat sampling, microfluidic transport, multibiofluid monitoring, continuous sweat-flow analysis, and integration with electrochemical point-of-care platforms [32,33,34]. More specialized reviews have addressed sweat glucose, electrolytes and pH, energy harvesting, data visualization, and artificial-intelligence-assisted interpretation of physiological signals [35,36,37,38]. These reviews provide valuable foundations, but they mainly ask what can be measured, how devices are built, or where they can be applied.

What remains less developed is a review logic centered on measurement distortion during exercise. This gap is especially important for potentiometric sweat electrolyte monitoring based on ISEs, because sample collection, microfluidic transport, ion recognition, ion-to-electron transduction, reference-potential stability, readout, transmission, and concentration interpretation are continuously coupled. A delay, mixing event, drift source, contamination pathway, or interpretation bias at one stage can propagate downstream and shift the final concentration estimate away from the current electrolyte state of sweat. Analyses focused only on materials, single electrodes, or broad application scenarios therefore cannot fully explain where measurement distortion originates, how it propagates, or which design and validation steps are needed during exercise.

**Figure 1:**
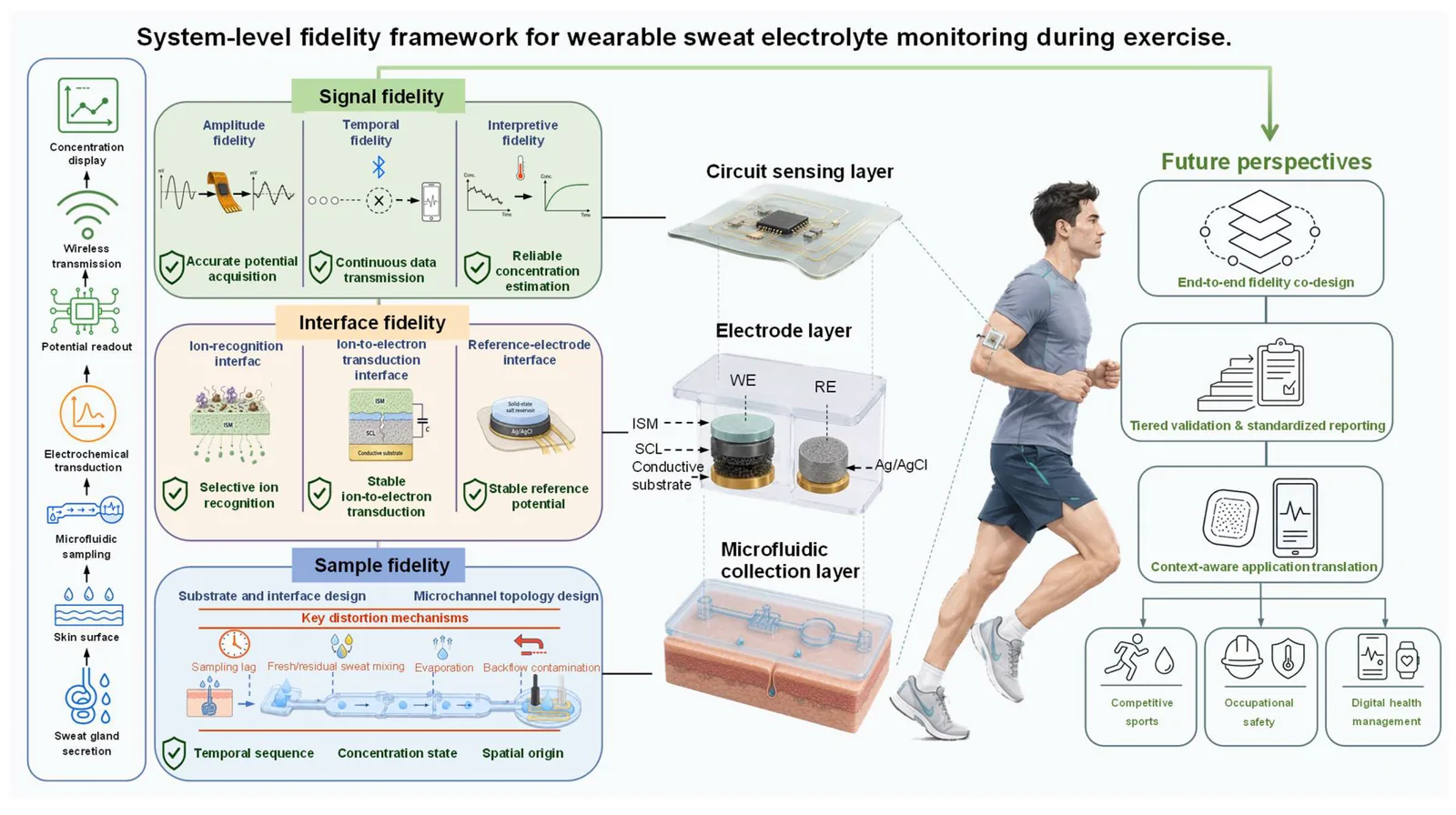
System-level fidelity framework for wearable sweat electrolyte monitoring during exercise.

To address this issue, this review uses fidelity as an organizing concept for wearable sweat electrolyte sensing. Fidelity is defined here as the ability of a sensing system to preserve the integrity, continuity, and interpretability of target sweat electrolyte information under real exercise conditions. We divide this problem into three coupled levels: sample fidelity, interface fidelity, and signal fidelity (Figure 1). Sample fidelity concerns whether sweat preserves its temporal sequence, concentration state, and spatial origin before reaching the sensing region. Interface fidelity concerns whether ion recognition, ion-to-electron transduction, and reference-potential output remain stable in complex sweat matrices under deformation. Signal fidelity concerns whether potential signals are accurately acquired, transmitted, corrected, and converted into concentration estimates. Together, these levels form a system-level fidelity chain from sweat generation to the reported electrolyte profile.

This review is scoped to wearable sweat electrolyte monitoring during exercise, with emphasis on potentiometric ISE-based systems for Na^+^, K^+^, and Cl^−^. We first discuss sample distortion during collection and transport, focusing on microfluidic substrates, wettability control, and microchannel topology. We then examine interface fidelity through three interfacial components: ISMs, solid-contact layers, and reference electrodes. Next, we analyze signal fidelity from electrode output to concentration interpretation, including readout, wireless transmission, calibration, compensation, and data correction. Finally, we summarize the design and validation requirements for system-level, high-fidelity sweat electrolyte monitoring under realistic exercise conditions.

## 2. Sample Fidelity

In wearable sweat electrolyte monitoring, whether sweat can reach the sensing region in a state close to its native condition is a fundamental prerequisite for measurements to accurately reflect physiological status [39]. Previous studies have shown that sweat retained on the skin surface or within the device may mix with newly secreted sweat and produce memory effects, causing the sensor output to deviate from the current state of sweat [20,40]. In other words, once sweat has undergone retention, mixing, evaporation, or backflow before reaching the sensing region, its original concentration and temporal information are generally difficult to fully recover, even with highly selective electrodes, low-noise circuits, or data-correction algorithms [34]. Sample fidelity should therefore be considered explicitly in microfluidic design for sweat electrolyte monitoring during exercise.

Sample fidelity can also be affected by conditions present before sweat enters the collection pathway. Sweat-induction mode influences the conditions under which a sample is generated: pharmacological stimulation with pilocarpine enables controlled local sweating at rest, whereas exercise-induced sweating is governed by thermoregulatory drive and may differ in sweat rate and electrolyte composition [4,5,41]. Initial skin conditions represent another source of pre-collection variability, because residual sweat and skin-surface mineral contamination can affect the composition of the first collected sample [42]. Standardized skin preparation and explicit reporting of sweat-induction and sampling conditions can therefore improve the comparability of sweat measurements across studies [4]. Exercise further complicates the preservation of sweat sample integrity. Sweat secretion rate varies continuously with exercise intensity [4], thermoregulatory responses, environmental conditions, and fluid intake, while stretching, bending, compression, and friction at the skin surface can continuously disturb sweat collection and transport [34]. From the perspective of sample fidelity, sweat information comprises three principal dimensions: time, concentration, and spatial origin. Temporal fidelity requires the sweat detected by the sensor to correspond as closely as possible to the current physiological state. Concentration fidelity requires the original electrolyte concentration to be preserved during collection, transport, and prior to detection, with minimal distortion from evaporation or mixing between fresh and residual sweat. Spatial-origin fidelity requires the sweat entering the sensing unit to originate predominantly from the target sampling region and to follow the intended transport pathway, thereby minimizing interference from sweat originating outside the target area or from backflow of waste fluid.

Accordingly, sample fidelity in this review refers to the ability to preserve the temporal sequence, concentration state, and spatial origin of sweat as it travels from secretion at the skin surface to the sensing region. To reduce distortion during this process, wearable sweat-sampling systems have progressed from simple collection layers and fluid-guiding channels toward designs that more actively regulate sweat entry, transport, renewal, separation, evaporation, and backflow. These advances involve complementary developments in substrate materials, skin-interfacing properties, wettability control, microchannel structures, and device encapsulation. Because this section focuses on the role of microfluidic systems in preserving and regulating the state of sweat samples, the following discussion is organized around two aspects: microfluidic substrate materials and microchannel topology. The former governs sweat entry, sample accommodation, and initial transport, whereas the latter further regulates sample renewal, temporal separation, evaporation, and backflow after sweat enters the device.

### 2.1. Microfluidic Substrate Materials

Sample fidelity is influenced by microfluidic substrate materials and their interfacial properties. As the first interface through which sweat enters the sensing system from the skin surface, the substrate serves not only as structural support and a means of device attachment but also directly determines whether temporal, concentration, or spatial-origin distortions occur before sweat reaches the sensing region [9]. Specifically, interfacial wettability affects the initiation of sweat entry under low sweat-rate conditions and therefore influences temporal fidelity. Pore structure, encapsulation, and resistance to evaporation affect sweat evaporation, retention, and lateral spreading during transport, thereby influencing concentration and spatial-origin fidelity [43]. Mechanical compliance and skin conformability also influence the stability of the skin–device sampling interface during movement [44,45]. Representative substrate and skin-interface designs combine enclosed transport, wettability control, and conformal attachment to improve sweat collection and transport before the sample reaches the sensing region [44,45,46,47].

**Figure 2:**
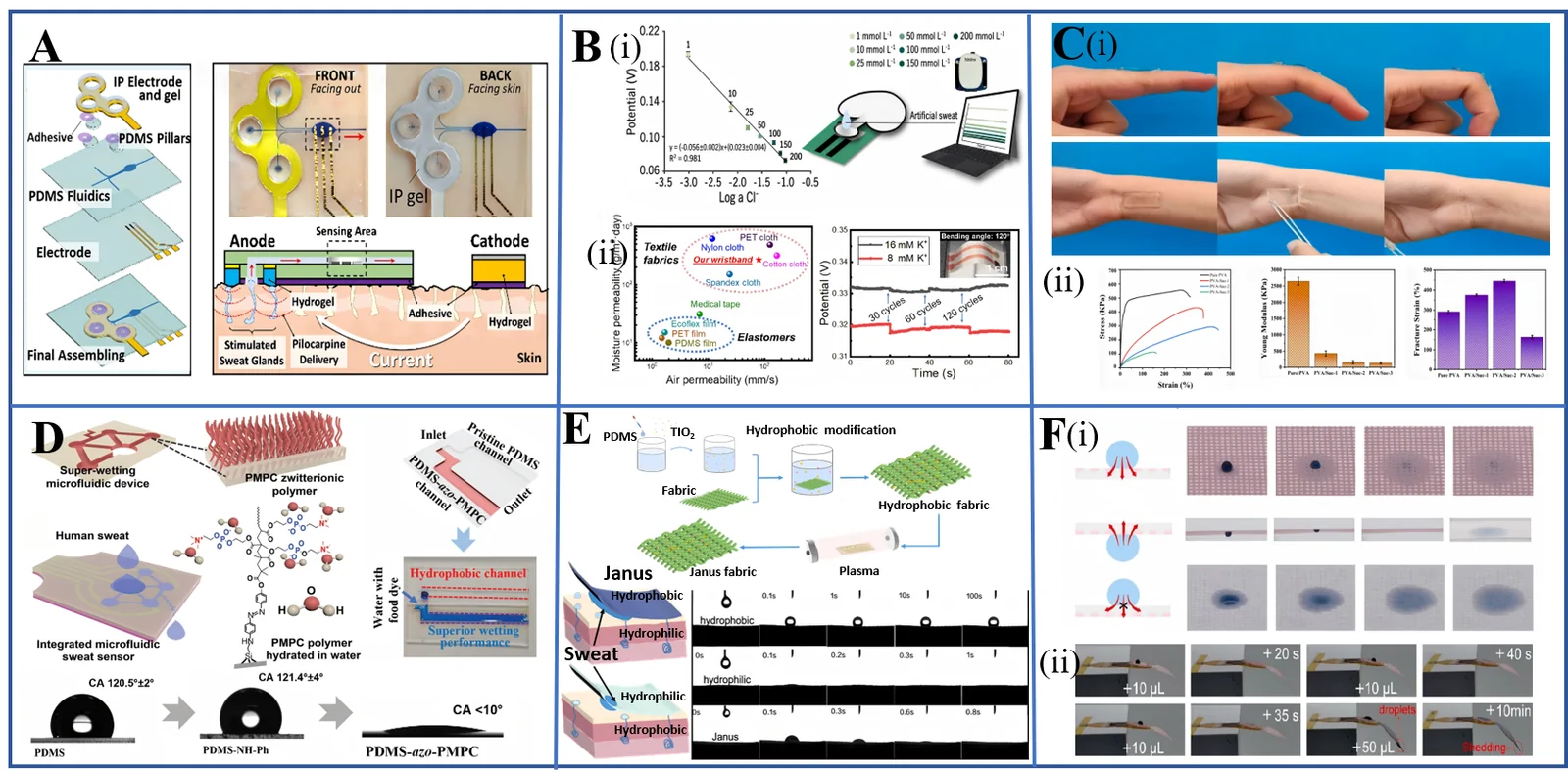
Microfluidic substrate materials and interfacial designs for sample fidelity. (**A**) Layered structure and sweat transport pathway of a PDMS-based enclosed sweat microfluidic device. Reproduced with permission: Copyright 2022, Springer Nature [46]. (**B**) Applications of fibrous-network substrates in low-sweat-rate collection and exercise wear: (**i**) a paper-based potentiometric sensor and its Cl^−^ calibration curve obtained with 10 μL artificial sweat, demonstrating the potential of paper-based materials for electrolyte detection in small sweat volumes. Reproduced with permission: Copyright 2026, Elsevier [54]; (**ii**) comparison of air/water-vapor permeability between a textile wristband and common flexible materials, together with the potential-response stability of a textile K^+^ sensor after repeated bending, demonstrating the wearing comfort and exercise adaptability of textile substrates. Reproduced with permission: Copyright 2023, Science Advances [44]. (**C**) (**i**) human fingers, demonstrating the ability of the hydrogel to maintain adhesion during dynamic movement, and hand skin, showing no visible skin irritation or residue upon removal. (**ii**) Mechanical properties of PVA-based hydrogels, including stress–strain curves, Young’s modulus, and fracture strain. Reproduced with permission: Copyright 2025, Elsevier [45]. (**D**) Superhydrophilic mechanism, contact-angle change, and channel-wetting behavior of PMPC-grafted PDMS. Reproduced with permission: Copyright 2021, John Wiley and Sons [47]. (**E**) Fabrication of asymmetric wettability in a Janus silk fabric, schematic illustration of sweat transport from the skin side, and unidirectional transport tests, showing the role of a hydrophilic/hydrophobic gradient in directing sweat collection. Reproduced with permission: Copyright 2023, Elsevier [55]. (**F**) (**i**) Images and schematic diagrams illustrating water-droplet permeation from the hydrophobic side, from the hydrophilic side, and anti-gravity transport from the hydrophobic side to the opposite side of the Janus paper. (**ii**) Flow process of blue ink on the Janus paper-based microfluidic patch. Reproduced with permission: Copyright 2026, Elsevier [56].

Dense polymer substrates are widely used in wearable sweat microfluidic devices, with representative materials including polydimethylsiloxane (PDMS), polyethylene terephthalate (PET), and photosensitive resins [20,43,48,49]. These materials generally offer high fabrication precision and effective encapsulation and can be used to construct well-defined microchannels through soft lithography, laser cutting, molding, or three-dimensional printing [49,50,51]. Owing to its flexibility, PDMS is commonly used to fabricate enclosed microchannels conformally attached to the skin (Figure 2A) [46]. Multilayer PET structures can be patterned by laser cutting and laminated to form planar microchannels [52], whereas printable polymers such as photosensitive resins facilitate the construction of vertical channels and complex three-dimensional fluid-guiding structures [53]. From the perspective of sample fidelity, the principal advantages of dense polymer substrates are their ability to improve the controllability and enclosure of sweat transport pathways, thereby reducing sample leakage, environmental contamination, and concentration shifts caused by evaporation from exposed surfaces. However, the limited intrinsic hydrophilicity of many polymer substrates can increase the resistance to sweat entry, particularly at low sweat rates. This limitation has been addressed through complementary strategies such as surface hydrophilization, inlet-geometry optimization, and shortened or vertically oriented transport pathways [47,53]. By combining well-defined enclosed channels with improved wetting and reduced transport resistance, these approaches retain the fabrication and encapsulation advantages of dense polymer substrates while reducing the delay associated with initial sweat collection.

Compared with dense polymer substrates, fibrous-network substrates, represented by paper and textiles, rely on their intrinsic porous structures and capillary action to reduce the resistance associated with sweat entry. Their porous structures and capillary transport can support sweat collection at low sweat rates or during the early stages of exercise [57,58,59]. Paper-based materials are inexpensive and readily patterned, and their capillary transport enables pump-free collection and introduction of small sweat volumes (Figure 2B(i)) [54]. Textile substrates provide compliance, breathability, and compatibility with movement, which can support attachment during prolonged exercise monitoring (Figure 2B(ii)) [44]. From the perspective of sample fidelity, fibrous-network substrates lower the threshold for initiating collection and thereby alleviate temporal distortion under low sweat-rate conditions. Nevertheless, their open pores and irregular fibrous pathways may also cause lateral spreading, increased dead volume, and mixing between fresh and residual sweat, thereby compromising concentration and spatial-origin fidelity [60]. Their application in sweat electrolyte monitoring therefore requires a balance among rapid collection, wearing comfort, and sample preservation.

Hydrogel substrates improve sample fidelity primarily by enhancing skin conformability and interfacial stability. Their high water content, mechanical compliance, and tissue-like mechanical properties help maintain stable device attachment to the skin, thereby reducing interfacial detachment and displacement of the sampling region during exercise [61]. For prolonged exercise monitoring, a stable skin–device interface helps preserve the consistency of the sampling region and thus improves spatial-origin fidelity. The hydrogel patch developed by Chenani et al. exhibited a low elastic modulus and high stretchability while maintaining adhesion during dynamic finger bending, demonstrating the advantages of hydrogel substrates in conformal attachment and interfacial stability (Figure 2C) [45]. However, hydrogels typically contain large amounts of water and possess swellable three-dimensional networks. After sweat enters the hydrogel, its absorption kinetics, changes in water content, and diffusion pathways may affect both the time required for analytes to reach the sensing interface and their concentration state, making the hydrogel itself a potential source of sample distortion [62]. Hydrogel substrates should therefore be evaluated not only in terms of adhesion, biocompatibility, and wearing comfort, but also with respect to sweat-absorption kinetics, ion-diffusion behavior, sample renewal time, and long-term interfacial stability [63].

In addition to the substrate material itself, hydrophilic–hydrophobic interfacial design is an important means of regulating initial sample fidelity. Hydrophilic modification can reduce inlet resistance in hydrophobic polymer microchannels and promote rapid sweat entry under low sweat-rate conditions, thereby mitigating temporal distortion caused by collection delay. Fu et al. grafted the zwitterionic polymer poly (2-methacryloyloxyethyl phosphorylcholine) (PMPC) onto PDMS surfaces through an azo-coupling reaction to produce superhydrophilic channels. This modification reduced the water contact angle from 121° to below 10° and markedly enhanced spontaneous wetting and fluid transport within the microchannels (Figure 2D) [47]. Janus structures with asymmetric wettability can establish hydrophilic–hydrophobic gradients that direct sweat unidirectionally from the skin-facing side toward the sensing side, thereby suppressing backflow contamination and improving spatial-origin fidelity [64]. Y. Zhang et al. produced a Janus silk fabric by applying PDMS/TiO_2_-based hydrophobic treatment to one side and plasma-induced hydrophilic treatment to the other. The resulting hydrophilic and hydrophobic surfaces exhibited contact angles of 39.5° and 130.5°, respectively, and demonstrated effective unidirectional liquid transport (Figure 2E) [55]. Patterned hydrophilic–hydrophobic interfaces can further restrict liquid spreading through hydrophobic boundaries and hydrophilic channels, thereby reducing uncontrolled diffusion, local retention, and inadequate drainage in porous substrates such as paper and textiles [60]. S. Zhang et al. developed a wax-printed Janus paper-based microfluidic patch in which patterned hydrophobic regions were formed by wax printing and combined with a wettability gradient to achieve directional liquid penetration and continuous drainage. Unidirectional transport tests showed that liquid could penetrate from the hydrophobic side to the hydrophilic side, whereas transport in the reverse direction was suppressed. Blue-dye flow experiments further demonstrated that liquid followed the predefined patterned pathway and was discharged through the outlet (Figure 2F) [56]. Importantly, the fidelity-preserving performance of hydrophilic–hydrophobic interfaces depends not only on their initial contact angles or single-cycle wetting behavior, but also on their long-term stability under sweat flushing, salt crystallization, skin friction, and repeated deformation [65]. Such interfaces should therefore be evaluated under dynamic transport, bending and stretching, and prolonged wearing conditions rather than solely on the basis of static wettability measurements.

Overall, advances in microfluidic substrates have progressively expanded the role of the skin-facing interface from passive mechanical support to active regulation of sweat collection and transport. Enclosed polymer substrates provide well-defined and protected transport pathways, whereas paper and textile networks exploit capillary transport to facilitate sweat entry at low secretion rates. Hydrogel-based interfaces improve conformal attachment and help maintain a stable sampling region during movement, while hydrophilic modification, patterned wettability, and Janus structures further regulate sweat entry, lateral confinement, and directional transport. Together, these approaches address different aspects of initial sample distortion by improving collection efficiency, sampling-site stability, and control over the direction and extent of sweat transport. After sweat enters the device, further preservation of sample renewal, temporal separation, evaporation resistance, and backflow control depends primarily on microchannel topology.

### 2.2. Microfluidic Topology

After the substrate material completes sweat collection and initial sample accommodation, microchannel topology further determines whether sweat can be continuously transported to the sensing region with minimal delay, residual volume, evaporation, and backflow. Existing designs are no longer limited to fluid guidance through a single channel. Instead, inlet arrays [66], branched channels [49], valve units [67,68], venting structures [69], and reset mechanisms [70] are used to regulate sweat entry, transport, separation, and discharge. Microchannels used during exercise should therefore be regarded not merely as passive fluid-guiding structures, but also as dynamic fluid-control units within the sample-fidelity chain [71]. Once sweat enters the device, these structures can further mitigate distortions arising from collection delay, mixing between fresh and residual sweat, evaporative concentration, and backflow contamination.

**Figure 3:**
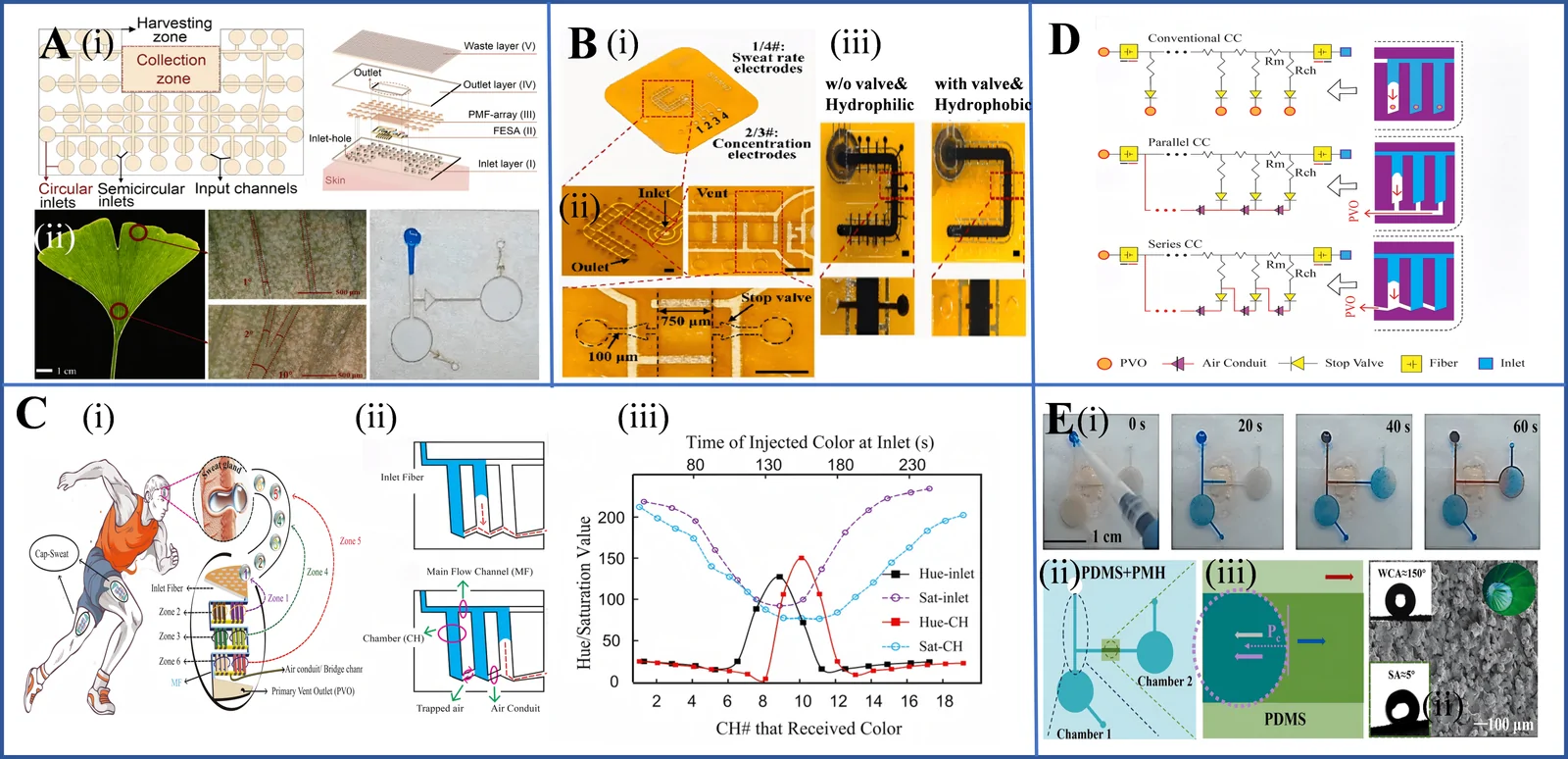
Representative microchannel-topology designs for sample fidelity. (**A**) Structures for suppressing collection delay: (**i**) a multi-inlet array increases the probability of sweat entry under low-sweat-rate conditions by enlarging the effective sampling area. Reproduced with permission: Copyright 2025, Elsevier [66]; (**ii**) a wedge-shaped channel inspired by ginkgo leaf veins enhances capillary driving force through a gradient geometry and shortens the effective transport path. Reproduced with permission: Copyright 2025, Elsevier [73]. (**B**) Structure for suppressing sweat retention: an adaptive resettable microchannel promotes channel emptying and repeated filling through sidewall vents, check valves, and hydrophobic liquid-limiting design, where (**i**) shows the overall structure, (**ii**) shows schematic illustrations of the sidewall vents and the structure with check valves, and (**iii**) compares structures with different check-valve/hydrophobic-treatment conditions. Reproduced with permission: Copyright 2024, Elsevier [74]. (**C**) Structure for suppressing temporal mixing: the Cap-Sweat platform achieves time-resolved sampling through segmented microchambers, preprogrammed capillary circuits, and a centralized venting network, where (**i**) shows the overall schematic of time-sequenced sweat collection during exercise, (**ii**) shows the capillary features used for time-resolved sampling, and (**iii**) shows the sequential conversion of dynamic dye inputs into temporally separated samples. MF, main flow capillary channel; PVO, vent outlet; CHs, microchambers. Reproduced with permission: Copyright 2026, ROYAL SOCIETY OF CHEMISTRY [75]. (**D**) Structure for suppressing evaporative concentration: a centrally vented capillary microfluidic design and its comparison with conventional multi-vent, parallel-vent, and serial-vent strategies; the right panel shows the corresponding channel layouts, where R_m_ denotes the hydraulic resistance of the main connecting channel and Rch denotes the hydraulic resistance of the branch channel. Reproduced with permission: Copyright 2026, ROYAL SOCIETY OF CHEMISTRY [75]. (**E**) Structure for suppressing backflow contamination: microfluidic valve and directional sweat-guiding designs, where (**i**) a wedge-shaped biomimetic channel combined with a superhydrophobic valve enables directional liquid transport, and blue and red dye solutions sequentially enter different sensing chambers, indicating reduced risks of backflow and mixing between fresh and residual samples; (**ii**) is a schematic of a wettability-gradient channel; and (**iii**) shows that the superhydrophobic region, with a high contact angle and low sliding angle, can serve as a local barrier to prevent unintended liquid migration. Reproduced with permission: Copyright 2025, Elsevier [76].

Collection delay under low sweat-rate conditions primarily compromises temporal fidelity. During the early stages of exercise or low-intensity activity, a simple straight channel with a single inlet often requires sweat to accumulate near the inlet to a threshold volume before transport begins. Consequently, the detected signal may lag behind the actual sweat-secretion process [72]. Microchannel topology is therefore commonly optimized by enlarging the effective sampling area, shortening the transport pathway, and reducing inlet flow resistance. Multi-inlet arrays enlarge the sampling area and increase the probability of sweat entering the device, thereby reducing sampling gaps caused by insufficient local sweat production (Figure 3A(i)) [66]. Vertical microchannels shorten the transport distance from the skin surface to the sensing region [53], whereas biomimetic collection structures use graded geometries or wedge-shaped channels to enhance capillary driving forces and direct sweat more rapidly toward the sensor (Figure 3A(ii)) [73]. The importance of these designs lies not merely in increasing the collected volume, but in lowering the minimum usable sweat rate, shortening the time required for sweat to reach the sensing region, and reducing inlet-initiation delay. These effects improve the temporal correspondence between the sampled sweat and the current exercise state, thereby enhancing sample fidelity.

Mixing between fresh and residual sweat caused by sample retention compromises concentration fidelity. In continuous sweat monitoring, sweat remaining in the substrate, microchannels, or sensing chamber may mix with newly secreted sweat and generate a memory effect, causing changes in electrolyte concentration to be averaged, delayed, or partially obscured [40]. To mitigate distortion arising from inadequate sample renewal, microchannel topology should minimize residence pathways, promote the discharge of residual sweat, and, where necessary, enable channel resetting [77]. Short transport pathways reduce the residence time of sweat within the device. Hashimoto et al. developed a short microfluidic pathway that rapidly directs sweat from the skin surface into a confined region to form quantifiable droplets, thereby reducing delays in sample renewal associated with retention in long channels [78]. M. Liu et al. designed an adaptive resettable microchannel incorporating sidewall vents, check valves, and hydrophobic liquid barriers to facilitate channel emptying and repeated filling. The sidewall vents reduce pneumatic resistance during liquid entry and discharge, whereas the check valves and hydrophobic treatment restrict unintended liquid retention and reverse migration (Figure 3B) [74].

In addition to residual sweat, insufficient separation of samples collected at different times during transport or storage can also cause sample mixing. During exercise, sweat electrolyte concentrations may change continuously with exercise intensity, thermoregulatory responses, and hydration status. If sweat from different time periods is mixed before reaching the sensing region or storage chamber, the resulting electrolyte signal may lose a clearly defined temporal label [20]. Segmented storage and time-resolved sampling have therefore become important strategies in microchannel-topology design. The Cap-Sweat platform uses segmented microchambers and preprogrammed capillary circuits to direct sweat sequentially into different chambers. Dynamic dye inputs were converted successively into temporally separated samples, demonstrating that this structure can preserve, to some extent, the order in which samples enter the device (Figure 3C) [75]. Although such designs are not equivalent to real-time continuous electrode measurements, their ability to time-stamp and segment sweat samples can improve the temporal fidelity of electrolyte-dynamics tracking during exercise.

Evaporative concentration primarily compromises concentration fidelity. Open channels, microchambers with multiple vents, and porous absorbent structures facilitate sweat entry, air displacement, and capillary uptake, but may also increase the area of contact between sweat and ambient air. Water loss through evaporation can consequently produce an apparently elevated electrolyte concentration [79]. Anti-evaporation topology must therefore balance efficient liquid entry against reduced environmental exposure [80]. Jalali et al. developed a centrally vented capillary microfluidic structure in which multiple microchambers were connected to a shared vent outlet through a venting network. The filling sequence was regulated through the hydraulic resistances of the main connecting channel and branch channels (Figure 3D) [75]. Compared with conventional multi-vent, parallel-vent, and serial-vent configurations, the centralized venting design reduces direct sample exposure to the external environment while allowing air-bubble removal and maintaining capillary filling, thereby helping to limit concentration distortion caused by evaporation.

Backflow contamination commonly occurs during compression, bending, and skin deformation associated with exercise. Previously measured sweat, sweat in the waste region, or sweat originating outside the target area may flow back into the sampling or sensing region under pressure disturbances, thereby compromising both spatial-origin and temporal fidelity. Valve structures, waste-isolation zones, and directional sweat-guiding layers are therefore commonly used to control reverse flow. Valves increase resistance to backward transport and prevent residual sweat from re-entering the sensing pathway [81,82]. Waste-isolation zones reduce the likelihood of contact between previously measured and freshly secreted sweat, whereas directional sweat-guiding layers or wettability-gradient structures direct sweat along a predefined pathway [83]. Liao et al. combined a wedge-shaped biomimetic channel with a superhydrophobic valve. The wedge-shaped structure enabled rapid directional transport, while the superhydrophobic valve separated sequentially introduced liquids into different sensing chambers, thereby reducing backflow (Figure 3E) [76].

Overall, microfluidic control in wearable sweat sensing has progressed beyond passive sweat routing toward more active regulation of sample transport and renewal. Multi-inlet and short-path structures reduce the delay between secretion and detection, resettable channels promote removal of residual sweat, segmented microchambers preserve the temporal order of sequential samples, centralized venting limits evaporative exposure, and valve or directional-transport structures restrict backflow. These approaches address different stages of the sampling pathway and together improve the temporal, concentration, and spatial-origin fidelity of sweat delivered to the sensing region. Because these strategies target different stages of the sampling pathway, their performance cannot be represented by collected volume or flow rate alone. Within the fidelity framework used in this review, Table 1 therefore summarizes sample-renewal time, transport delay, evaporative loss, residual volume, and resistance to reverse flow as complementary evaluation dimensions.

## 3. Interface Fidelity

Sample fidelity ensures that sweat reaches the sensing region in a state close to its native condition; however, this is only a prerequisite for accurate monitoring. In potentiometric sweat electrolyte sensors, changes in ionic activity must be recognized by the ion-selective membrane, converted from ionic to electronic signals through the solid-contact layer, and measured against a stable reference potential before they can be translated into interpretable potential signals [84]. If these interfacial processes are disturbed by the complex sweat matrix, prolonged wetting, or skin deformation, the sensor may still exhibit responses unrelated to the target ions even when the sweat sample itself retains satisfactory temporal, concentration, and spatial-origin fidelity. Interface fidelity therefore serves as the critical link between sample fidelity and signal fidelity.

In this review, interface fidelity refers to the ability of a potentiometric sweat electrolyte sensor to maintain stable ion recognition, low-drift ion-to-electron transduction, and a reliable reference potential under real exercise and wearing conditions. Based on the operating processes of solid-contact ion-selective working electrodes and patch-type solid-state or quasi-solid-state reference electrodes, interfacial distortion can be classified into three categories. The first is recognition-interface distortion, which primarily arises from the adsorption of sweat-derived contaminants on the ion-selective membrane and from the migration, redistribution, or loss of ionophores, plasticizers, and ion exchangers within the membrane [85,86]. These processes may lead to reduced selectivity, delayed response, or changes in the calibration curve. The second is transduction-interface distortion, which mainly results from the formation of an aqueous layer beneath the membrane, insufficient capacitance of the solid-contact layer, and interfacial instability under mechanical deformation [87,88,89]. Its manifestations include open-circuit potential drift, amplified noise, and irreversible responses. The third is reference-interface distortion, which primarily originates from depletion of the salt reservoir, instability of the liquid-junction interface, and variations in the composition of the external sample [90]. These effects can cause fluctuations in the reference potential and may be misinterpreted as changes in target-ion concentration. Accordingly, this section discusses the distortion mechanisms, fidelity-preserving strategies, and evaluation methods associated with the recognition, transduction, and reference interfaces.

### 3.1. Recognition-Interface Fidelity

Recognition-interface fidelity depends on whether the ion-selective membrane (ISM) can maintain stable interfacial ion exchange, selective recognition, and membrane-phase composition during exposure to complex sweat, prolonged wetting, and repeated deformation [84]. Recognition distortion mainly arises from two sources. The first is surface fouling, in which proteins, lipids, skin debris, and other sweat- or skin-derived components adsorb onto the membrane surface and alter the mass transfer, wetting, and partitioning of target ions at the sweat–membrane interface [85]. The second is internal compositional drift, in which plasticizers, ionophores, or ion exchangers migrate, redistribute, or leach during prolonged wetting and continuous sweat renewal, thereby altering membrane composition and the number of effective recognition sites [86]. Accordingly, this section discusses recognition-interface fidelity during exercise-related sweat monitoring from two perspectives: resistance to surface fouling and stability of the internal membrane composition.

**Figure 4:**
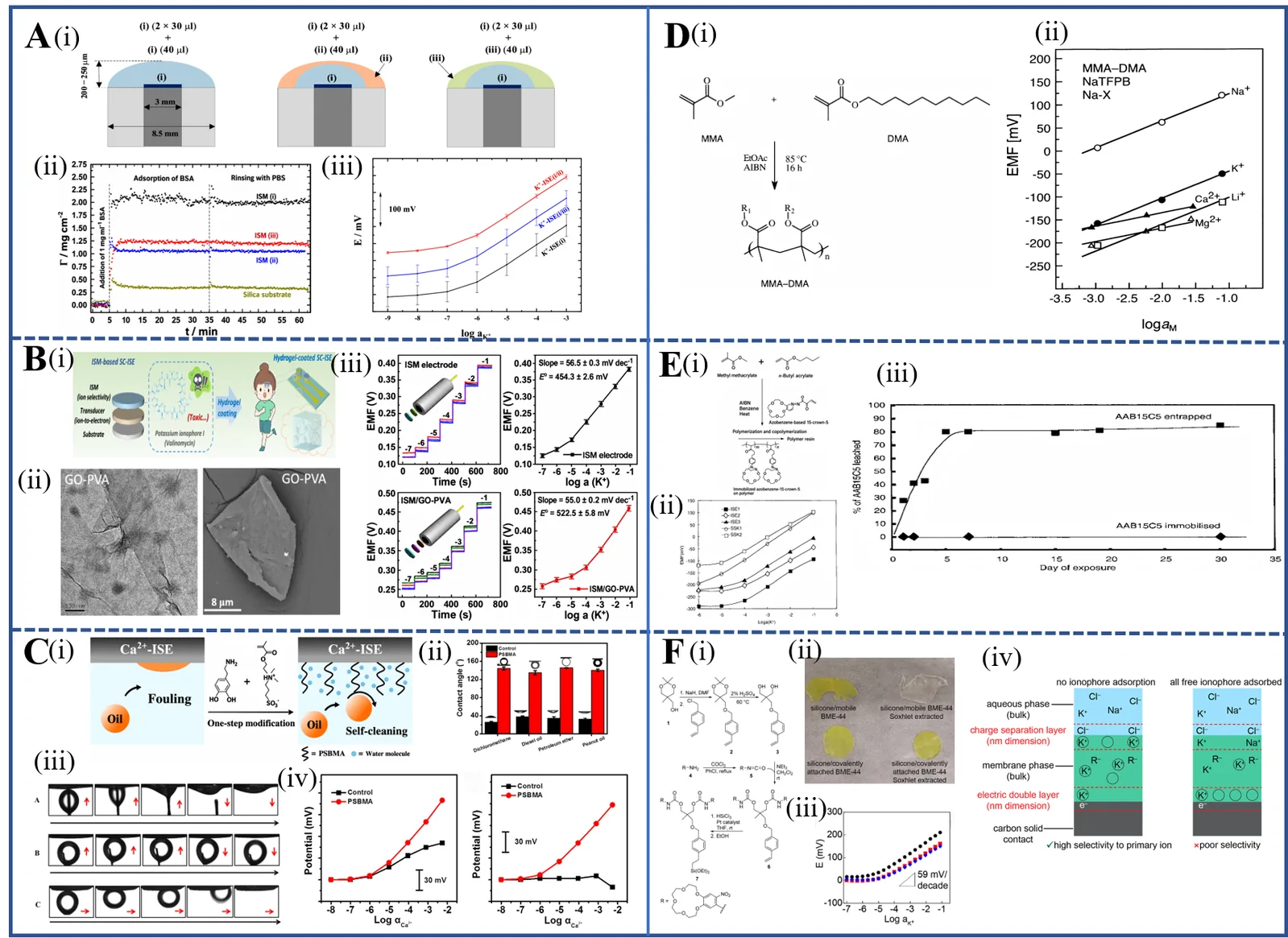
Antifouling and compositional-stability designs of ISMs for preserving ion-recognition interface fidelity. (**A**) (**i**) Schematic illustration of the outer-layer structure of SR, (**ii**) comparison of bovine serum albumin (BSA) adsorption, and (**iii**) comparison of E^0^ reproducibility. Reproduced with permission: Copyright 2019, AMERICAN CHEMICAL SOCIETY [91]. (**B**) (**i**) Typical structure of an ISM-based solid-contact ion-selective electrode (SC-ISE), (**ii**) wearable SC-ISE with a graphene oxide–poly (vinyl alcohol) (GO–PVA) hydrogel coating for sweat K^+^ monitoring, together with transmission electron microscopy (TEM) and scanning electron microscopy (SEM) images of the GO–PVA hydrogel, and (**iii**) potential responses and calibration curves of the ISM and ISM/GO–PVA electrodes. Reproduced with permission: Copyright 2016, Springer Nature [92]. (**C**) (**i**) Preparation of the zwitterionic polymer-modified membrane, (**ii**) schematic illustration of the antifouling mechanism of the modified Ca^2+^-ISE against oil contamination, (**iii**) comparison of underwater oil contact angles before and after modification, and (**iv**) self-cleaning performance of the poly (sulfobetaine methacrylate) (PSBMA)-modified ISM and calibration curves before and after modification under oil-contaminated conditions. Reproduced with permission: Copyright 2021, AMERICAN CHEMICAL SOCIETY [93]. (**D**) (**i**) Schematic synthesis of a plasticizer-free methyl methacrylate–decyl methacrylate (MMA–DMA) copolymer matrix and (**ii**) potentiometric response function in solution. Reproduced with permission: Copyright 2002, John Wiley and Sons [94]. (**E**) (**i**) Schematic illustration of the one-step immobilization of the 15-crown-5 ionophore in a self-plasticized methacrylate–acrylate copolymer, (**ii**) comparison of leaching curves between embedded and immobilized ionophores, and (**iii**) K^+^ response performance. Reproduced with permission: Copyright 2000, AMERICAN CHEMICAL SOCIETY [95]. (**F**) (**i**) Synthesis of a covalently anchorable BME-44 potassium-ionophore derivative, (**ii**) comparison of free and covalently anchored BME-44 membranes before and after Soxhlet extraction, (**iii**) K^+^ potentiometric response curves, and (**iv**) schematic illustration of ionophore adsorption induced by a high-specific-surface-area carbon solid-contact layer and the resulting ionophore depletion from the membrane phase. Reproduced with permission: Copyright 2024, AMERICAN CHEMICAL SOCIETY [96].

To mitigate surface fouling, dense polymer outer layers, protective hydrogel coatings, and zwitterionic hydration layers have been employed to reduce contaminant adsorption and maintain a stable recognition environment at the membrane surface. A dense polymer outer layer can modify the properties of the outermost ISM surface and reduce direct contact between proteinaceous contaminants and the ionophore-containing membrane phase. Joon et al. introduced a silicone rubber (SR) outer layer onto a poly (vinyl chloride) (PVC)-based K^+^-selective membrane. This layer reduced the adsorption of bovine serum albumin (BSA) and improved standard-potential reproducibility without compromising electrode selectivity (Figure 4A) [91]. Such strategies reduce nonspecific adsorption through interfacial isolation; however, the response may still depend on redistribution of plasticizers, ionophores, and lipophilic ion exchangers from the inner membrane into the outer layer. The coating therefore does not function as a completely inert physical barrier. Hydrogel protective layers place greater emphasis on hydrophilicity and biocompatibility. S. Liu et al. coated the ISM of a K^+^-selective solid-contact ion-selective electrode (K^+^-SC-ISE) with a graphene oxide–poly (vinyl alcohol) (GO–PVA) hydrogel. The coating prevented direct contact between the skin and the ISM while preserving a near-Nernstian response and ion selectivity (Figure 4B) [92]. However, the hydrogel layer may increase the ion-diffusion distance. Its thickness, pore structure, and mechanical stability must therefore be balanced against the competing requirements of antifouling protection and rapid response. Zwitterionic coatings reduce the adhesion of hydrophobic contaminants by forming strongly hydrated interfacial layers, providing a molecular-level strategy for suppressing contamination by lipids and sebum in sweat (Figure 4C) [93]. Although the cited study did not specifically investigate sweat, its mechanism for suppressing the adhesion of hydrophobic contaminants may inform interfacial protection against lipid- and sebum-related fouling in sweat sensing.

Beyond surface fouling, recognition fidelity also depends on the stability of the internal membrane composition. Conventional PVC-based ISMs rely on the physical blending of plasticizers, ionophores, and ion exchangers, and their selective responses depend on stable component ratios and a consistent number of effective recognition sites within the membrane. Prolonged exposure to sweat, continuous sample renewal, and changes in skin temperature may promote the migration, redistribution, or leaching of functional membrane components, leading to reduced slopes, altered selectivity, and poorer response repeatability. One strategy for addressing this problem is to employ plasticizer-free or self-plasticizing polymer matrices, which can reduce the loss of externally added plasticizers while preserving membrane flexibility and ion-transport properties. Qin et al. prepared plasticizer-free ISMs using a methyl methacrylate–decyl methacrylate (MMA–DMA) copolymer, which exhibited favorable potentiometric responses and selectivity for different ion systems (Figure 4D) [94]. Building on this approach, Heng et al. immobilized ionophores within a self-plasticizing polymer network. Leaching experiments showed that the immobilized ionophores exhibited lower losses than physically entrapped ionophores, while the resulting membranes retained their potentiometric response to K^+^ (Figure 4E) [95]. Nevertheless, ion exchangers and other functional additives may remain mobile, and ionophore immobilization may alter intramembrane mobility, membrane resistance, and response kinetics. Covalent anchoring provides a more direct molecular strategy for suppressing ionophore loss. Spindler et al. used plasticizer-free silicone rubber as the membrane matrix and synthesized a BME-44 potassium-ionophore derivative bearing triethoxysilane groups, allowing the ionophore to become incorporated into the crosslinked network during silicone condensation curing. Dichloromethane Soxhlet extraction showed that up to approximately 96% of the ionophore could be immobilized within the silicone membrane. Compared with membranes containing free BME-44, membranes containing the covalently anchorable BME-44 derivative retained a Nernstian response to K^+^ and exhibited improved K^+^/Na^+^ selectivity and lower membrane resistance (Figure 4F(i–iii)) [96]. However, covalent anchoring is not free from engineering limitations. If the reactive BME-44 derivative contacts a high-surface-area carbon solid-contact layer during silicone curing, it may preferentially adsorb onto the carbon surface. This can deplete the effective ionophore concentration in the membrane phase and reduce the selectivity of the solid-contact ISE (Figure 4F(iv)) [96]. The effectiveness of covalent immobilization therefore depends not only on whether the ionophore can be incorporated into the polymer network, but also on curing conditions, ionophore solubility, membrane–electrode interfacial materials, and the fabrication sequence. For sweat electrolyte monitoring during exercise, an ideal ISM should exhibit satisfactory Nernstian response and selectivity during initial calibration while also maintaining stable membrane-component ratios, recognition-site density, and membrane-phase mechanical properties during prolonged sweat exposure, repeated deformation, and extended wear [97].

Overall, strategies for preserving the recognition interface have progressed from optimizing the initial ion-selective response toward maintaining both the membrane surface and its internal composition during prolonged use. Protective polymer and hydrogel layers, together with zwitterionic surface modification, reduce direct exposure of the ISM to fouling species, whereas plasticizer-free matrices, ionophore immobilization, and covalent anchoring reduce the mobility and loss of functional membrane components. These approaches address complementary sources of recognition drift and improve the stability of the ion-recognition environment beyond initial calibration. Within the fidelity framework used in this review, recognition-interface evaluation therefore extends beyond initial sensitivity and selectivity to response stability after contaminant exposure, prolonged wetting, and repeated deformation.

### 3.2. Transduction-Interface Fidelity

Solid-contact ion-selective electrodes (SC-ISEs) replace the conventional inner-filling solution with a solid-contact layer, which facilitates device miniaturization, flexibility, and system integration. However, eliminating the inner-filling solution also weakens the conventional stable ionic-buffering environment, making the ion-to-electron transduction process more dependent on the stability of the ISM, the solid-contact layer, and their buried interface. For wearable sweat electrolyte monitoring during exercise, continuous sweat wetting, fluctuations in ion flux, and repeated mechanical perturbations such as bending, stretching, and friction may all affect the transduction interface, causing drift, hysteresis, or noise amplification during the conversion of ion activity into potential output. Transduction-interface fidelity is therefore a key component of interface fidelity, and its core objective is to maintain a stable, low-drift, and recoverable ion-to-electron conversion process.

Distortion at the transduction interface can be broadly classified into three categories. The first is the formation of an aqueous layer beneath the membrane, where water penetrates the ISM and accumulates between the membrane and the solid-contact layer, creating a liquid interfacial phase that may induce baseline drift and response hysteresis [87]. The second is insufficient interfacial capacitance. According to the relationship between capacitance and potential fluctuation, ΔE = ΔQ/C, limited charge-storage capacity in the solid-contact layer can amplify small charge perturbations into open-circuit potential fluctuations and noise [88]. The third is mechanical instability under deformation, where repeated bending, stretching, and friction can cause membrane cracking, delamination of the contact layer, or damage to the conductive network, making it difficult for the electrode to maintain stable transduction under dynamic wearing conditions [89]. Correspondingly, fidelity-preserving strategies for the transduction interface can be developed from three aspects: suppression of the aqueous layer, enhancement of interfacial capacitance, and stabilization of the mechanical structure.

**Figure 5:**
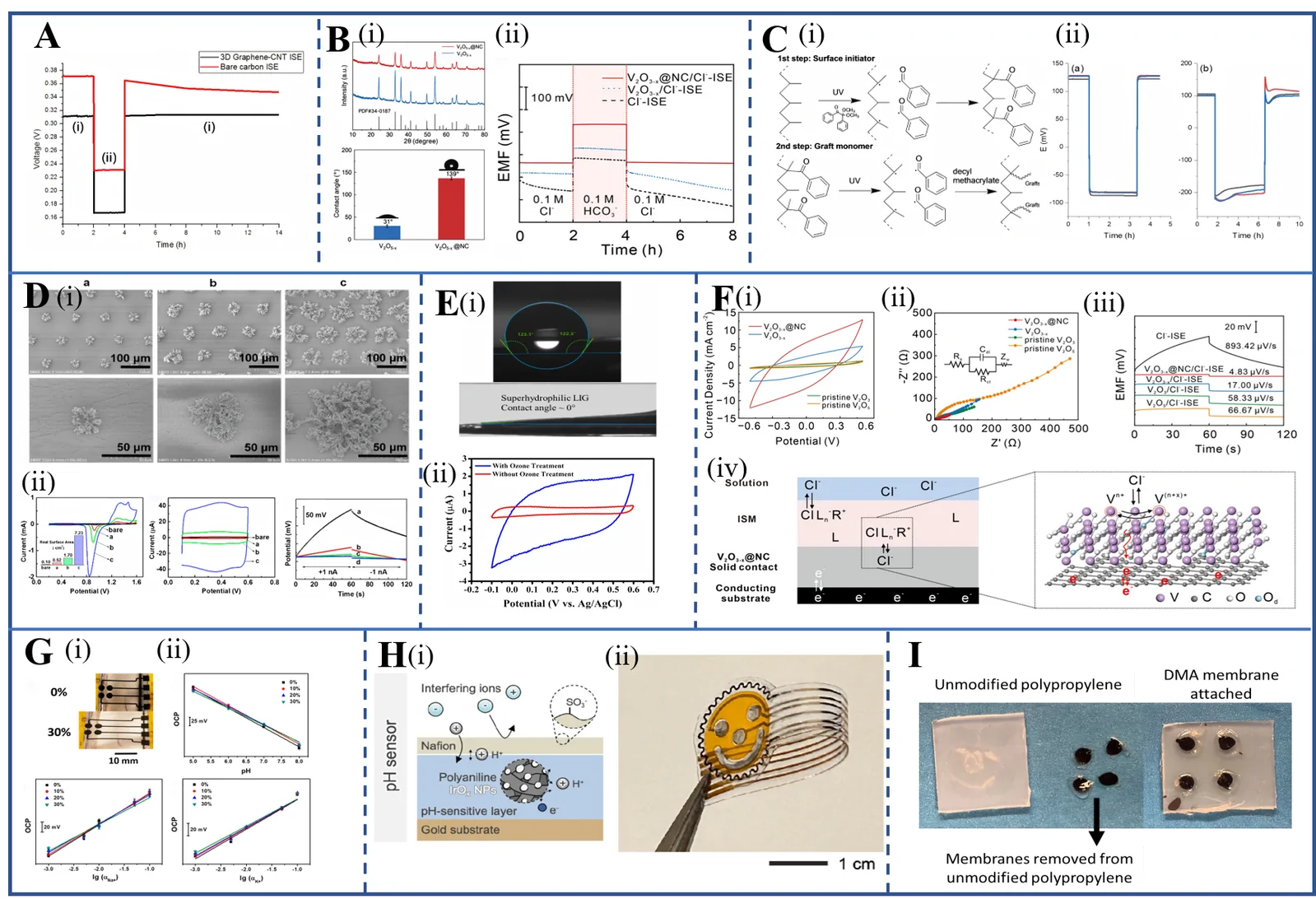
Solid-contact layers and membrane/electrode interface designs for transduction-interface fidelity. (**A**) Aqueous-layer test comparison between 3D graphene–CNT-modified electrodes and bare carbon electrodes. Reproduced with permission: Copyright 2023, MDPI [98]. (**B**) (i) Structural characterization and water contact angles of V_2_O_3−x_ and V_2_O_3−x_@NC, and (**ii**) aqueous layer test results. Reproduced with permission: Copyright 2025, American Chemical Society [99]. (**C**) (**i**) Reaction schematic of covalent membrane/electrode interface construction via photo-initiated graft polymerization, and (**ii**) aqueous-layer test comparison between covalently and noncovalently connected electrodes. Reproduced with permission: Copyright 2023, John Wiley and Sons [100]. (**D**) (**i**) SEM images of gold nanodendrite arrays prepared with different deposition times, where a, b, and c correspond to 60, 180, and 300 s, respectively; (**ii**) cyclic voltammetry curves, effective surface area estimation, and chronopotentiometric curves. Reproduced with permission: Copyright 2017, **American Chemical Society** [101]. (**E**) (**i**) Water contact angle images of pristine LIG and O-LIG, and (**ii**) cyclic voltammetry curves. Reproduced with permission: Copyright 2021, Elsevier [102]. (**F**) (**i**) Cyclic voltammetry curves, (**ii**) electrochemical impedance spectra, (**iii**) chronopotentiometric curves, and (**iv**) schematic illustration of the interfacial transduction mechanism of the V_2_O_3−x_@NC pseudocapacitive solid-contact layer and comparison materials. Reproduced with permission: Copyright 2025, American Chemical Society [99]. (**G**) (**i**) Stretchable v-AuNW ion-selective electrode, and (**ii**) potential responses under different tensile strains. Reproduced with permission: Copyright 2020, **American Chemical Society** [103]. (**H**) (**i**) Biphasic PANI/IrO_x_ pH-sensing interface, and (**ii**) photograph of the flexible device. Reproduced with permission: Copyright 2025, **American Chemical Society** [104]. (**I**) Comparison of peel tests for covalently anchored and noncovalently connected membrane/electrode interfaces. Reproduced with permission: Copyright 2023, John Wiley and Sons [100].

To address aqueous-layer formation, common strategies include constructing hydrophobic solid-contact layers, applying hydrophobic coatings to intrinsically hydrophilic transduction materials, and strengthening the interfacial adhesion between the ISM and the solid-contact layer. Hydrophobic carbon materials are representative choices for reducing the risk of interfacial water storage. Hua et al. introduced a three-dimensional graphene–carbon nanotube (3D graphene–CNT) composite network into ISEs, using the rough three-dimensional carbon network and its hydrophobic characteristics to enhance resistance to water accumulation at the ISM/electrode interface. Aqueous-layer tests showed that the 3D graphene–CNT-modified electrode exhibited a more stable potential output than the bare carbon electrode, indicating that the hydrophobic carbon network reduced the risk of aqueous-layer formation at the membrane/electrode interface (Figure 5A) [98]. For solid-contact materials that are intrinsically hydrophilic, surface hydrophobic coating is also an effective strategy. C.-L. Wang et al. constructed a nitrogen-doped carbon coating on the hydrophilic V_2_O_3−x_ transduction material, increasing its water contact angle from 31° to 139°. Aqueous-layer tests showed that the V_2_O_3−x_@NC electrode exhibited a more stable potential response, indicating that the outer hydrophobic carbon coating helped reduce interfacial water accumulation (Figure 5B) [99]. Compared with directly selecting hydrophobic carbon materials, this strategy is significant because it modifies transduction materials that are otherwise prone to water storage, providing a design principle based on “core functionalization–outer hydrophobic protection” for future solid-contact layers. Aqueous-layer formation may also be associated with microvoids between the ISM and the solid-contact layer. Interfaces formed solely through physical adsorption may undergo local delamination during prolonged wetting or mechanical perturbation, thereby creating space for water insertion. To address this issue, K.R. Choi et al. used photo-initiated graft polymerization to connect the ISM simultaneously to a high-specific-surface-area carbon contact layer and an inert polymer substrate. Aqueous-layer tests showed that, compared with the ungrafted interface, the grafted interface exhibited lower potential drift, indicating that chemical anchoring can reduce the risk of aqueous-layer formation beneath the membrane (Figure 5C) [100].

To address insufficient interfacial capacitance, solid-contact layers are commonly designed to increase the effective interfacial area, improve membrane/electrode contact, and introduce pseudocapacitive mechanisms to enhance charge-buffering capacity. S. Wang et al. constructed gold nanodendrite arrays (AuNDs), in which highly branched hierarchical micro/nanostructures increased the real surface area of the electrode and thereby enhanced the double-layer capacitance of the solid-contact layer. By tuning the AuND deposition time to obtain solid-contact layers with different surface areas, they demonstrated that AuNDs with larger surface areas improved charge-storage capacity and potential stability (Figure 5D) [101]. Surface-chemical regulation can further improve membrane-solution infiltration and the actual transduction area. Choudhury et al. used laser-induced graphene (LIG), and ultraviolet–ozone (UV–ozone) treatment adjusted its surface wettability and introduced oxygen-containing functional groups, allowing the ISM cocktail to infiltrate the porous LIG electrode more effectively. As shown by the electrochemical tests before and after treatment (Figure 5E) [102], the areal capacitance of ozonized LIG (O-LIG), estimated from cyclic voltammetry curves, increased from 4.8 to 42.4 μF/cm^2^. This strategy does not simply increase the external geometric area; rather, it improves the effective area actually involved in transduction by enhancing membrane infiltration and interfacial contact. Furthermore, introducing pseudocapacitive materials can provide additional charge-storage pathways beyond double-layer capacitance, thereby enhancing the interfacial charge-buffering capacity. The V_2_O_3−x_@NC solid-contact layer constructed by C.-L. Wang et al. enhanced charge storage through the synergistic effects of oxygen vacancies, multivalent vanadium centers, and the nitrogen-doped carbon layer. The multivalent vanadium centers provided reversible valence-transition sites, whereas the nitrogen-doped carbon layer promoted electron transport. Electrochemical tests showed that V_2_O_3−x_@NC exhibited higher interfacial capacitance, lower charge-transfer resistance, and lower potential drift (Figure 5F) [99]. These results indicate that pseudocapacitive mechanisms can enhance the ability of the solid-contact layer to buffer charge perturbations, thereby reducing open-circuit potential fluctuations.

To address mechanical instability, fidelity-preserving design of the transduction interface should improve deformation adaptability through structural stretchability, rigid–soft composite buffering, and interfacial chemical anchoring [100,103,104]. At the structural level, Zhai et al. developed a stretchable ion-to-electron transduction layer based on vertically aligned gold nanowires and applied it to pH-, Na^+^-, and K^+^-selective electrodes. The vertically aligned gold nanowire (v-AuNW) structure was embedded in an elastic PDMS substrate and maintained conductive pathways and electrochemical responses under stretching, allowing the sensors to retain relatively stable potential responses under different tensile strains (Figure 5G) [103]. This finding indicates that the coordinated design of nanowire arrays and elastic substrates can reduce deformation-induced fracture of conductive networks and instability of interfacial contact. At the material-composite level, flexible polymer layers can act as stress-buffering and interfacial-anchoring units for rigid inorganic materials. Lee et al. constructed a biphasic PANI/IrO_x_ pH-sensing interface, in which the electrochemically polymerized PANI layer provided flexible support, conductive pathways, and interfacial adhesion, while the IrO_x_ nanoparticles supplied the response sites (Figure 5H) [104]. This “flexible polymer underlayer–inorganic oxide response-particle” composite design helps relieve stress concentration in rigid transduction materials on flexible substrates and reduces the risk of local delamination or mechanical instability during hydration and deformation. At the interfacial-chemistry level, covalent anchoring can further enhance the resistance of the buried membrane/electrode interface to delamination. K.R. Choi et al. connected the ISM simultaneously to a high-specific-surface-area carbon contact layer and an inert polymer substrate through photo-initiated graft polymerization. Peel tests showed that the membrane and solid-contact layer on the unmodified substrate were readily removed under external force, whereas the covalently connected membrane/solid-contact layer formed after surface modification retained better adhesion (Figure 5I) [100]. These results indicate that covalent anchoring can reduce the risk of membrane delamination under prolonged wetting, thermal stress, and mechanical deformation, thereby improving the mechanical fidelity of the transduction interface.

Overall, solid-contact design has progressed from simply replacing the inner-filling solution toward coordinated control of interfacial hydration, charge buffering, and mechanical integrity. Hydrophobic contact layers and strengthened membrane/electrode adhesion reduce aqueous-layer formation, high-surface-area and pseudocapacitive materials improve charge-storage capacity and potential stability, and stretchable conductive networks, compliant composite structures, and covalent anchoring help maintain interfacial continuity during deformation. These developments address different origins of transduction instability and together improve the ability of solid-contact ISEs to maintain low-drift and mechanically stable potential responses. Within the fidelity framework used in this review, these improvements are therefore considered in terms of their stability during prolonged wetting, repeated deformation, and dynamic sweat exposure, rather than initial Nernstian response and short-term stability alone.

### 3.3. Reference-Interface Fidelity

For exercise-oriented sweat monitoring, mechanical deformation and repeated wet–dry cycling further increase the need to evaluate whether the reference potential can recover after transient perturbation. Reference-electrode selection should therefore consider not only initial potential stability but also monitoring duration, sweat composition, hydration conditions, electrolyte loss, and mechanical robustness.

Potentiometric sensors measure the potential difference between the working electrode and the reference electrode; therefore, stability of the reference potential directly affects the reliability of the final readout. In wearable sweat monitoring, electrolyte leakage, external-sweat ingress, changes in liquid-junction composition, depletion of the internal salt reservoir, and repeated wetting or deformation may all perturb the reference interface and produce potential shifts unrelated to the target-ion concentration [90]. This issue is particularly relevant to Cl^−^ monitoring because Cl^−^ is both the target analyte and a species involved in the equilibrium of conventional Ag/AgCl reference electrodes. In this review, reference-interface fidelity therefore refers to the ability of a reference electrode to maintain a stable baseline potential under realistic exercise and wearing conditions.

**Figure 6:**
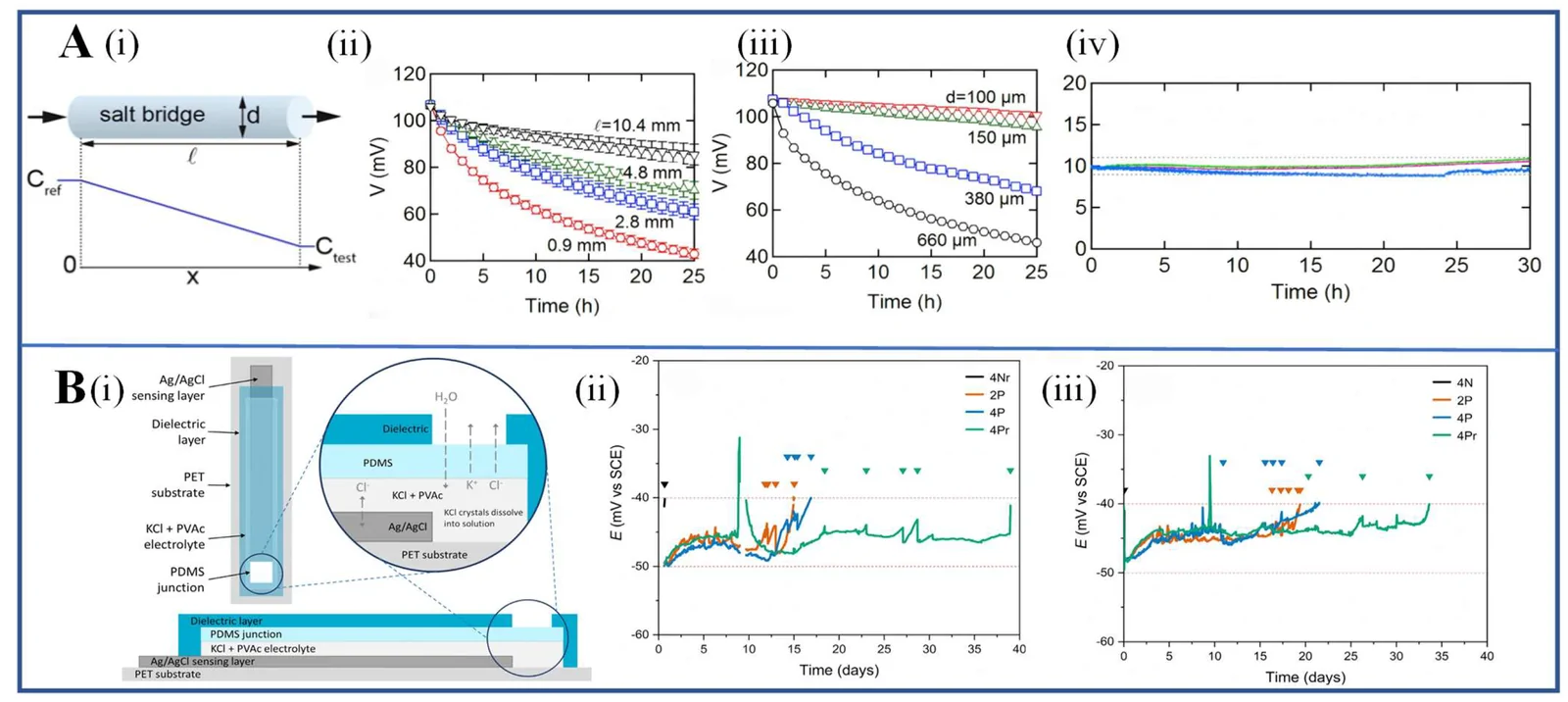
Solid-state and quasi-solid-state reference-electrode designs for preserving reference-interface fidelity. (**A**) Salt-bridge geometry and diffusion-path regulation. (**i**) Salt-bridge geometry controls the equilibrium between the reference solution and the test solution; time-dependent potential changes under different (**ii**) salt-bridge lengths and (**iii**) salt-bridge diameters; and (**iv**) concentration stability within 30 h after salt-bridge optimization. Reproduced with permission: Copyright 2016, **American Chemical Society** [105]. (**B**) Salt-containing polymer reservoirs and confined liquid-junction membrane regulation. (**i**) Schematic structure of a screen-printed Ag/AgCl reference electrode with a KCl/PVAc electrolyte layer and a PDMS liquid-junction membrane; (**ii**,**iii**) comparison of open-circuit potential stability among reference electrodes with different structures, showing that the electrode incorporating the KCl/PVAc electrolyte layer and PDMS liquid-junction membrane exhibits lower drift and a longer stable lifetime during long-term testing. Reproduced with permission: Copyright 2021, Elsevier [108].

Existing miniaturized reference electrodes mainly preserve reference stability by regulating electrolyte exchange or reducing dependence on freely diffusible internal electrolytes. Salt-bridge and liquid-junction designs control the diffusion path between the internal electrolyte and external sweat. D.-H. Choi et al. showed that increasing salt-bridge length reduced the time-dependent potential decay of a wearable Cl^−^ sensor, whereas increasing bridge diameter accelerated drift, demonstrating the importance of junction geometry (Figure 6A) [105]. Planar liquid-junction structures have also been incorporated into fully screen-printed Cl^−^ sensors, combining reference-potential stabilization with a fabrication format compatible with compact wearable integration [106]. A related approach is to immobilize concentrated electrolyte within hydrogel or polymer matrices. Hydrogel/KCl reservoirs have been incorporated into flexible sweat patches for continuous Cl^−^ monitoring during exercise [107]. KCl/PVAc electrolyte layers combined with liquid-junction membranes have also been used to maintain a local Cl^−^ reservoir while restricting electrolyte loss (Figure 6B) [108], whereas NaCl-containing PVB layers have been employed to reduce the sensitivity of Ag/AgCl reference electrodes to changes in the external ionic environment [22].

Reference interfaces that rely less strongly on aqueous salt reservoirs provide another route toward improved long-term stability and miniaturization. Ionic-liquid-based reference membranes have been incorporated into flexible wearable platforms for Na^+^ and K^+^ monitoring during exercise [109], and more recent planar designs have demonstrated ionic-liquid-containing polymer membranes compatible with printed electrode fabrication [110]. Low-leakage solid-state reference electrodes further reduce material exchange by replacing highly soluble internal electrolytes with sparingly soluble salts or immobilized ionic phases. Gan et al. developed an Ag/AgTPB/PVC-TBATPB reference electrode in which the reference potential was governed primarily by the Ag/AgTPB–TPB^−^ interfacial equilibrium rather than by a concentrated soluble KCl reservoir. The electrode exhibited relatively small potential variations under changes in electrolyte composition and showed favorable stability during water-layer and long-term soaking tests [111].

These approaches offer complementary advantages but also introduce different limitations. Geometry-controlled liquid junctions and immobilized salt reservoirs remain susceptible to gradual electrolyte depletion, whereas ionic-liquid and low-leakage solid-state interfaces require further evaluation of matrix sensitivity, material aging, interfacial water-layer formation, and fabrication reproducibility.

Within the fidelity framework used in this review, reference-interface evaluation extends beyond short-term open-circuit potential stability to potential variation under different ionic strengths, pH values, temperatures, and Cl^−^ backgrounds; long-term drift during continuous wetting; stability during wet–dry cycling and dynamic sweat renewal; and potential recovery after bending or compression. Salt-bridge regulation, immobilized electrolyte reservoirs, ionic-liquid reference membranes, and low-leakage solid-state interfaces together provide complementary strategies for reducing reference-induced baseline distortion and improving the reliability of wearable potentiometric sweat monitoring.

Overall, advances in potentiometric interface design have addressed instability at the recognition, transduction, and reference interfaces through complementary material and structural strategies. Antifouling surface layers and stabilized membrane compositions improve the persistence of ion recognition; hydrophobic, high-capacitance, and mechanically robust solid-contact interfaces improve ion-to-electron transduction; and controlled liquid junctions, immobilized electrolyte reservoirs, ionic-liquid membranes, and low-leakage solid-state designs improve reference-potential stability. Together, these developments have moved wearable ISE design beyond optimization of initial sensitivity and selectivity toward preservation of interfacial stability during continuous operation. Remaining limitations are increasingly associated with the durability of these improvements under complex sweat exposure, prolonged wetting, repeated deformation, and device-to-device fabrication variability. Table 2 summarizes the major interfacial distortion mechanisms, corresponding fidelity-preserving strategies, and evaluation metrics discussed in this section.

## 4. Signal Fidelity

Sample fidelity and interface fidelity address the preservation of sweat sample integrity and the stability of electrode-interface responses, respectively. However, the final monitoring outcome also depends on whether the potential signal can be accurately acquired, continuously recorded, and appropriately interpreted [112]. In potentiometric sweat electrolyte sensors, once changes in target-ion activity have been converted into a potential difference, the signal must subsequently undergo analog front-end readout, analog-to-digital conversion, wireless transmission, data correction, and conversion from potential or ion-activity responses into concentration estimates [14,113]. Problems such as insufficient input impedance, electrical noise, data loss, inadequate temperature compensation, or mismatch between the calibration model and actual measurement conditions may introduce errors into the final concentration estimates, even when sample and interface fidelity are well preserved [13].

In this review, signal fidelity refers to the ability to preserve the amplitude, temporal sequence, and interpretive relationship of the potential signal as accurately, continuously, and traceably as possible from electrode output to the generation of concentration estimates. This section examines signal fidelity from three perspectives: amplitude fidelity, temporal fidelity, and interpretive fidelity. Amplitude fidelity concerns signal perturbations during potential readout and analog-to-digital conversion. Temporal fidelity concerns data integrity during continuous recording, time synchronization, and wireless transmission. Interpretive fidelity concerns the effects of temperature compensation, drift correction, and concentration conversion on the reliability of the reported results. These sources of signal distortion have been progressively addressed through high-impedance and low-noise readout circuits, miniaturized and wireless electronics, improved data-recording architectures, and calibration and compensation strategies for concentration estimation [114,115,116,117,118,119,120,121,122].

### 4.1. Amplitude Fidelity

Amplitude fidelity refers to the ability to preserve the original magnitude and variation pattern of a potentiometric sensor output during front-end readout, analog-to-digital conversion, and system integration. Ion-selective electrodes, particularly those incorporating high-resistance ion-selective membranes, can be represented as voltage sources with high source impedance and should therefore be measured under conditions approaching an open circuit [114,115,123]. If the input impedance of the front-end circuit is insufficient relative to the electrode source impedance, finite loading may introduce voltage-divider errors. Input bias currents and other leakage currents may also generate additional voltage drops across the high-impedance electrode and disturb the near-zero-current equilibrium at the electrode interface, causing the recorded signal to deviate from the open-circuit potential difference between the working and reference electrodes. For a monovalent ion, the ideal Nernstian slope at 25 °C is approximately 59.16 mV per decade [113]; accordingly, a potential error of 1 mV corresponds to an approximately 4% relative error in ion activity inferred from the Nernst equation. Although the actual error also depends on the electrode slope, concentration range, and calibration model, this estimate demonstrates that millivolt-level readout errors can substantially affect the final concentration result.

For high-impedance potentiometric signals, AFEs with high input impedance, low input bias current, and low noise are used to minimize electrical loading and preserve the electrode open-circuit potential [114,115].

Analog-to-digital conversion and sampling further affect the transformation of analog potential outputs into digital data. Appropriate analog-to-digital converter (ADC) resolution, a stable reference voltage, and suitable averaging strategies can help preserve millivolt-level potential variations and reduce random fluctuations. Cazalé et al. [116] used a portable electronic module to acquire potentiometric signals in a sweat Na^+^ monitoring system and combined analog-to-digital conversion with sample averaging to obtain stable outputs. However, the nominal bit depth of an ADC does not necessarily represent the effective resolution of the complete acquisition system, which is also limited by front-end noise, reference-voltage fluctuations, and other disturbances along the signal chain [117]. Within the fidelity framework used in this review, ADC performance is therefore considered in terms of effective potential resolution, noise, and acquisition stability rather than nominal bit depth and sampling frequency alone [117].

Alongside improvements in front-end readout, wearable sweat-sensing electronics have become progressively more compact and integrated. Early fully integrated platforms combined flexible sensor arrays with on-body signal processing and wireless communication, reducing reliance on benchtop instrumentation [15]. Further developments have incorporated miniature wireless readout circuits into wearable microfluidic platforms and have integrated sensing [24], signal processing, Bluetooth transmission, power management, and motion-based energy harvesting within a battery-free wearable system [23].

Flexible electronic integration provides a structural basis for maintaining amplitude fidelity during exercise. Li et al. developed a portable potentiometric detection system based on a flexible printed circuit board and a low-power Bluetooth module. Integration with a microfluidic electrochemical sensor and a smart terminal enabled on-body monitoring of sweat electrolytes, and the measurements obtained using the portable module were compared with those from a commercial electrochemical workstation [118]. Ma et al. integrated a K^+^-selective sensor, voltage-conditioning circuitry, and a wireless communication module into a textile wristband. Interconnect encapsulation and conductive-pattern design improved the mechanical stability and water resistance of the system, enabling continuous sweat K^+^ monitoring during exercise [44]. Together, these studies show that electronic miniaturization and flexible integration can move potentiometric readout from laboratory instrumentation toward self-contained wearable systems while maintaining on-body signal acquisition during exercise [44,118].

Overall, advances in wearable readout electronics have reduced the dependence of potentiometric sweat sensors on bulky laboratory instrumentation by combining high-impedance signal acquisition, analog-to-digital conversion, flexible circuitry, wireless communication, and increasingly compact power and transmission modules. High-input-impedance and low-noise front ends preserve millivolt-level electrode signals, while portable and flexible electronic integration enables continuous acquisition during movement and sweat exposure. Within the fidelity framework used in this review, amplitude-fidelity evaluation therefore considers front-end input characteristics, effective potential resolution, baseline noise, long-term drift, and agreement with reference instrumentation.

### 4.2. Temporal Fidelity

Temporal fidelity refers to the ability of potentiometric signals to preserve their true temporal order and dynamic relationships during continuous acquisition, data transmission, and recording. During exercise, sweat electrolyte concentrations may vary continuously with exercise intensity, sweat rate, thermoregulatory responses, and fluid intake. If sampling intervals are unstable, data transmission is interrupted, or timestamps are inaccurate, the resulting concentration profile may become misaligned with the actual stages of exercise, even when the potential measured at each individual time point is accurate. Such temporal misalignment can compromise the interpretation of dynamic monitoring results.

Existing wearable sweat-monitoring systems employ different data-recording and transmission architectures. For example, Ozer et al. integrated a K^+^-selective electrode, a miniature potentiometric readout circuit, and a Wi-Fi communication module into a wearable platform, enabling real-time transmission, display, and storage of the acquired data on a smart terminal [119]. In contrast, the sweat-monitoring system developed by Huang et al. first digitized the signal using a 12-bit analog-to-digital converter embedded in a microcontroller and continuously stored the acquired data in local memory. The data were subsequently retrieved and displayed through near-field communication (NFC) when a mobile terminal was brought close to the device [120]. The former architecture uses continuous wireless streaming after acquisition, whereas the latter relies on continuous local recording followed by intermittent wireless readout. Both architectures support continuous data acquisition or result visualization, but they differ in the temporal relationships among acquisition, buffering, transmission, and terminal updating. Therefore, describing a system as “real-time” or “continuous” is not, by itself, sufficient to characterize its temporal fidelity.

From the perspectives of hardware and data-link performance, temporal distortion mainly arises from two sources. First, an insufficient sampling frequency reduces temporal resolution and may cause short-lived changes to be missed, whereas irregular sampling intervals disrupt the true temporal relationship between adjacent data points [124]. Second, packet loss, buffering delays, connection interruptions, or terminal-refresh latency during wireless transmission may introduce gaps or temporal shifts into the concentration profile [125,126]. This section focuses on temporal distortion arising from electronic acquisition and data transmission. Although sample renewal time and sensor-interface response time belong primarily to sample fidelity and interface fidelity, respectively, they must still be distinguished from electronic acquisition and wireless transmission delays when interpreting the final monitoring curve.

At the acquisition stage, stable sampling intervals and a common time base help preserve temporal alignment among continuously recorded signals [124,127]. At the recording and transmission stages, local data storage, timestamp synchronization, and data-alignment methods can improve continuity and temporal correspondence when wireless communication is intermittent [120,125,126]. These measures help distinguish distortion introduced during electronic acquisition and communication from delays originating in sweat sampling or sensor-interface response.

Overall, wearable data architectures have evolved beyond simple real-time display toward more flexible combinations of continuous acquisition, local storage, wireless streaming, and intermittent readout [119,120]. Continuous wireless transmission enables immediate visualization, whereas local buffering and memory can preserve acquired data when communication is intermittent. Timestamping, synchronized acquisition, and data-alignment methods further improve the temporal correspondence between signals recorded by wearable systems [125,126,127]. Within the fidelity framework used in this review, temporal-fidelity evaluation therefore considers sampling stability, transmission latency, data completeness, and timestamp or multichannel alignment accuracy.

### 4.3. Interpretive Fidelity

Interpretive fidelity refers to the ability to convert acquired and recorded potential signals into sweat electrolyte concentrations through appropriate calibration, compensation, and experimental validation. Ion-selective electrodes respond directly to the activity of the target ion, and their potential is approximately proportional to the logarithm of ion activity according to the Nernst relationship. However, most studies report the concentrations of ions such as Na^+^, K^+^, and Cl^−^. The conversion from activity-dependent responses to concentration estimates depends not only on the response slope, but also on the calibration intercept, activity coefficient, device-to-device fabrication variability, and sample matrix [128].

Multipoint calibration is currently one of the most commonly used approaches for concentration conversion. A series of standard solutions with known concentrations is typically used to establish a potential–log concentration calibration curve, after which the ion concentration in an unknown sample is calculated using the measured slope and intercept [113]. Compared with the direct use of the theoretical Nernstian slope, multipoint calibration reflects the actual response characteristics of an individual electrode and can partially compensate for variations in initial potential among devices. Nevertheless, multipoint calibration does not guarantee that the calibration relationship remains valid throughout the entire wearing period; its applicability still depends on the stability of the electrode response during use.

The continued validity of a calibration relationship is mainly affected by the standard potential, response slope, and baseline drift. If the standard potential or baseline changes with wetting time, the intercept of the initial calibration curve may gradually become invalid. If the response slope changes, the potential–concentration relationship will likewise deviate from its initial state. Rousseau et al. [122] identified standard-potential reproducibility and electromotive-force drift as important indicators of calibration stability in all-solid-state ion-selective electrodes. Improving the consistency of the standard potential among devices from the same batch and reducing long-term drift can help minimize calibration variability. However, a response slope close to the theoretical value is not, by itself, sufficient to ensure reliable calibration-free sensing. Although calibration-free operation or reduced calibration frequency is an important future objective, its applicability to real sweat during exercise requires rigorous validation [129].

Temperature variation also affects potential-to-concentration conversion. On the one hand, the Nernstian response slope depends on absolute temperature. On the other hand, ion transport within the membrane, interfacial conditions, and reference-electrode stability may also change with temperature. Skin and device temperatures are not constant during exercise; therefore, when a calibration curve established at a fixed temperature is used throughout monitoring, part of the measured potential variation may reflect temperature effects rather than changes in ion concentration. Shiwaku et al. integrated a temperature-sensing unit into a wearable system and applied temperature-dependent corrections to Na^+^, K^+^, and pH signals, demonstrating that synchronous temperature measurement can support potential-to-concentration conversion [121]. The study by Shiwaku et al. further demonstrates that experimentally determined temperature-correction relationships can account for temperature-dependent changes in sensor response beyond the theoretical Nernstian slope alone [121].

The complex composition of real sweat further limits the direct application of calibration relationships established in standard solutions. Ion-selective electrodes respond to activity, whereas ionic strength, coexisting ions, and organic constituents in real sweat can all affect electrode output [130]. Consequently, obtaining a near-Nernstian response in a single-salt solution or artificial sweat does not necessarily demonstrate equivalent concentration accuracy during human exercise. To address this issue, some studies have compared wearable-sensor measurements with ion chromatography, commercial ion analyzers, or laboratory electrochemical methods. Parrilla et al. emphasized the importance of real-sweat testing and standard analytical methods in validating wearable potentiometric ion sensors [12]. Such comparisons help evaluate the entire analytical chain, from electrode response and portable acquisition to concentration conversion.

Beyond calibration and compensation, post-acquisition filtering, anomaly detection, and signal smoothing can be used to reduce random fluctuations or identify obvious outliers. G. Liu et al. applied Savitzky–Golay filtering, Z-score-based detection, and wavelet denoising to nonstationary electrochemical signals, providing useful approaches for wearable-data processing [131].

Taken together, concentration interpretation has progressed from simple conversion using a fixed Nernstian relationship toward calibration and validation procedures that account for device-specific response, temporal drift, temperature variation, and real-sweat matrix effects [113,121,122,130]. Multipoint calibration improves device-specific concentration conversion, drift control helps preserve calibration validity over time, synchronous temperature sensing enables compensation for thermal effects, and comparison with standard analytical methods provides an external check on the final concentration estimate. Signal filtering and anomaly detection can further improve data quality when used without obscuring genuine physiological variations. Despite these advances, differences in calibration matrices, compensation procedures, validation methods, and error reporting still limit direct comparison among wearable systems [132]. The interpretive fidelity discussed here is limited to the conversion of potentiometric signals into sweat electrolyte concentrations. Whether sweat electrolyte concentrations can subsequently represent whole-body hydration status, blood electrolyte levels, or individual fluid-replacement requirements is a separate question influenced by sweat rate, sampling location, environmental temperature, and interindividual variability [133,134].

Overall, advances in signal acquisition, electronic integration, data transmission, and concentration interpretation have progressively reduced distortion between electrode output and the reported electrolyte profile. High-impedance and low-noise electronics improve preservation of potential amplitude; miniaturized, flexible, and wireless systems enable continuous on-body acquisition; buffering, timestamping, and transmission-control strategies improve temporal integrity; and calibration, temperature compensation, drift control, and reference-method validation improve concentration interpretation. These developments demonstrate that signal fidelity increasingly depends on coordinated design of hardware, communication, and data-processing stages rather than on any single electronic or computational component. Table 3 summarizes the major sources of signal distortion, their corresponding mitigation strategies, and relevant evaluation metrics.

## 5. Conclusions and Perspectives

This review frames wearable sweat electrolyte monitoring during exercise as a system-level fidelity problem. The proposed sample, interface, and signal fidelity framework shifts attention from isolated electrode metrics to the preservation of electrolyte information across the full measurement chain. Sample fidelity determines whether sweat retains its temporal sequence, concentration state, and spatial origin before reaching the sensing region. Interface fidelity determines whether ion recognition, ion-to-electron transduction, and reference-potential output remain stable in complex sweat matrices and under deformation. Signal fidelity determines whether potential outputs are accurately acquired, transmitted, corrected, and converted into concentration estimates. This framework provides a practical structure for identifying where distortion is introduced and how it propagates into the reported electrolyte profile.

For device design, the main implication is that fidelity must be engineered across linked modules rather than added after electrode optimization. Sampling delays can disturb temporal alignment; residual-sweat mixing and evaporation can bias concentration; interfacial fouling or component leaching can appear as electrolyte changes; reference drift can shift the baseline; and readout, transmission, or calibration errors can amplify upstream uncertainty. System design should therefore integrate sampling, transport, recognition, transduction, reference stabilization, encapsulation, acquisition, transmission, and interpretation as an interdependent chain. Optimizing a single material, channel, electrode, or algorithm is insufficient if the neighboring stages remain unvalidated.

For performance evaluation, standard-solution electrode metrics should be treated as an entry point rather than proof of on-body reliability. Future studies should report sample renewal time, evaporative loss, backflow resistance, wettability stability, reference-potential stability, post-deformation recovery, wireless data integrity, temperature-compensation accuracy, and concentration-estimation uncertainty. A tiered validation pathway should connect standard solutions, artificial sweat, collected human sweat, and on-body exercise studies. This pathway would help distinguish intrinsic electrode behavior from sample-handling errors, interfacial instability, electronic readout errors, and physiological interpretation limits. As part of this validation framework, signal-processing algorithms should support rather than substitute for transparent calibration and validation, and excessive smoothing should be avoided when it could obscure physiologically meaningful signal variations.

The physiological interpretation of sweat electrolyte data should remain deliberately bounded. Reliable Na^+^, K^+^, and Cl^−^ concentration estimates are necessary, but they are not sufficient for determining whole-body electrolyte status, blood electrolyte levels, dehydration severity, or individualized fluid-replacement prescriptions. Sweat electrolyte concentrations are influenced by sweat rate, sampling location, exercise intensity, heat load, humidity, acclimation, interindividual variability, and fluid intake. Future studies should therefore integrate high-fidelity electrolyte estimates with sweat rate, skin temperature, heart rate, workload, environmental exposure, and individual baseline data before making physiological or behavioral recommendations.

With these boundaries, high-fidelity wearable sweat electrolyte monitoring could support sports, occupational safety, and digital health. In sport, it may help contextualize fluid replacement, training load, and recovery monitoring. In high-temperature or physically demanding workplaces, it may contribute to heat-stress risk assessment when combined with workload and environmental data. In digital health, it may provide complementary biofluid information for exercise safety and longitudinal self-monitoring. The field should therefore move from demonstrating attractive devices under standardized conditions toward reporting end-to-end fidelity under realistic exercise use. The sample, interface, and signal fidelity framework offers one route toward more reliable design, evaluation, and translation.

## Figures and Tables

**Table 1:** Major distortion mechanisms, fidelity-preserving strategies, and suggested evaluation metrics for sample fidelity.

Distortion Mechanism	Fidelity Dimension	Major Causes	Consequences	Fidelity-Preserving Strategies	Suggested Evaluation Metrics
Initial sampling variability	Temporal/concentration	Sweat-induction mode, residual sweat, skin-surface contamination	Variable sweat onset/rate, initial sample, composition bias	Controlled induction conditions [5,41], standardized skin preparation [42]	Sweat-onset time ^b^, sweat rate ^a^; first-sample deviation ^c^, stabilization time ^c^
Sampling lag	Temporal	Low sweat rate, high inlet resistance, long transport path	Signal lag	Hydrophilic inlets [47], multi-inlet arrays [66], short/vertical or gradient channels [53,73]	Initiation time ^b^, transit time ^b^
Fresh-residual sweat mixing	Temporal/concentration	Dead volume, sample retention, poor drainage	Temporal averaging, attenuated concentration changes	Low-dead-volume/short-path channels [77,78], resettable channels, venting/drainage [74]	Sample-renewal time ^b^, residual-volume fraction ^c^, concentration-switching response time ^b^
Temporal overlap of sequential samples	Temporal/concentration	Incomplete sample separation, unclear renewal interval	Loss of temporal identity, mixed concentration profiles	Segmented chambers, sequential sampling, programmed capillary routing [75]	Temporal resolution ^b^, sample interval ^b^
Evaporative concentration	Concentration	Open structures, multiple vents, insufficient encapsulation	Water loss, apparent concentration increase	Enclosed channels, centralized venting [75], low-evaporation encapsulation [80]	Evaporative loss rate ^b^, concentration retention ratio ^c^
Backflow contamination	Spatial-origin/temporal	Compression/deformation, reverse-flow pathways, poor waste isolation	Residual/waste-sweat re-entry, source contamination	Directional transport [76,83], superhydrophobic/check valves [76,81,82], waste isolation [83]	Reverse-pressure threshold ᵇ, post-deformation recovery time ^c^

^a^ metrics supported by established standards or methodologies; ^b^ metrics reported in previous studies but not yet standardized; ^c^ metrics proposed or further systematized in this review and requiring further methodological validation.

**Table 2:** Major interfacial distortion mechanisms, fidelity-preserving strategies, and suggested evaluation metrics for interface fidelity.

Interfacial Component	Major Distortion Risks	Resulting Potential-Response Deviations	Fidelity-Preserving Strategies	Suggested Evaluation Metrics
Recognition interface: membrane surface	Protein/lipid adsorption, skin-derived fouling	Delayed response, reduced selectivity, E^0^ drift	Antifouling outer layers [91], hydrogel protection [92], zwitterionic coatings [93]	Response time ^a^, selectivity coefficient ^a^, *E*^0^ reproducibility, post-fouling slope retention ᵇ
Recognition interface: membrane bulk	Ionophore/plasticizer/ion-exchanger leaching or redistribution	Calibration drift, attenuated long-term response	Plasticizer-free membranes [94], self-plasticizing matrices/ionophore immobilization [95], covalent anchoring [96]	Long-term slope retention ^b^, component-leaching rate ^b^, selectivity retention ^c^
Transduction interface: membrane/solid contact	Interfacial water-layer formation	OCP drift, delayed response	Hydrophobic solid contacts/coatings [98,99], interfacial grafting/anchoring [100]	Water-layer test ^a^, OCP drift rate ^a^
Transduction interface: charge buffering	Low interfacial capacitance	Noise amplification, baseline fluctuation	High-surface-area contacts [101], improved interfacial contact [102], pseudocapacitive materials [99]	Interfacial capacitance ^a^, impedance parameters ^a^, chronopotentiometric resistance/capacitance/drift
Transduction interface: mechanical stability	Cracking, delamination, conductive-network disruption	Irreversible drift, signal interruption	Stretchable conductive networks [103], stress-buffering layers [104], interfacial anchoring [100]	Post-deformation slope retention ^b^, potential-drift change ^b^, response-recovery time ^c^
Reference interface	Electrolyte depletion/leakage, unstable liquid junction, external-ion interference	Reference-potential drift; apparent analyte changes	Salt-bridge geometry [105]; planar liquid junctions [106]; hydrogel/polymer salt reservoirs [22,107]; ionic-liquid interfaces [109,110]; low-leakage solid-state reference [111]	Reference-potential drift rate ^a^, external-ion sensitivity ^b^, operational lifetime ^b^, wet–dry cycling stability ^c^

^a^ metrics supported by established standards or methodologies; ^b^ metrics reported in previous studies but not yet standardized; ^c^ metrics proposed or further systematized in this review and requiring further methodological validation.

**Table 3:** Major signal-distortion sources, fidelity-preserving strategies, and suggested evaluation metrics for signal fidelity.

Fidelity Dimension	Distortion Sources	Output Deviations	Fidelity-Preserving Strategies	Suggested Evaluation Metrics
Amplitude	Front-end loading, bias/leakage current, circuit/ADC noise	Potential error, concentration-estimation bias	High-input-impedance/low-bias AFE [114,115,123], low-noise acquisition, stable ADC reference [116,117]	AFE input impedance ^a^, input bias current, RMS potential noise ^a^, effective potential resolution ^a^
Temporal	Low/irregular sampling rate, transmission latency, packet loss, timestamp drift	Missed transients, data gaps, temporal misalignment	Stable sampling/timestamping [124,127], local buffering [120], packet-loss/data-integrity controls [125,126]	Sampling frequency ^a^; transmission latency ^a^; packet-loss rate ^a^; data completeness ^c^; timestamp/multichannel alignment error ^b^
Interpretive	Calibration mismatch, temperature effects, drift, matrix effects	Concentration-conversion bias, unreliable estimates	Multipoint/matrix-aware calibration [113,128], drift control [122,129], temperature compensation [121], real-sweat/reference-method validation [12,130,132], outlier detection [131]	Calibration bias/RMSE ^a^; drift rate ^a^; pre-/post-compensation error ^b^; agreement with standard methods ^a^

The superscript classifications of the evaluation metrics are the same as those defined in the note to Table 1. AFE, analog front end; ADC, analog-to-digital converter; RMSE, root-mean-square error. ^a^ metrics supported by established standards or methodologies; ^b^ metrics reported in previous studies but not yet standardized; ^c^ metrics proposed or further systematized in this review and requiring further methodological validation.

## Data Availability

No new data were created or analyzed in this study. Data sharing is not applicable to this article.
